# White matter matters in β-glucocerebrosidase-related pathologies

**DOI:** 10.4103/NRR.NRR-D-25-00158

**Published:** 2025-09-29

**Authors:** Loris Russo, Matilde Cescon

**Affiliations:** Department of Molecular Medicine, University of Padova, Padova, Italy

**β-glucocerebrosidase in health and disease:** Mutations in the β-glucocerebrosidase (*GBA*) gene do cause the rare lysosomal storage disorder Gaucher’s disease (GD) with an estimated global prevalence of 1:200,000 (Imbalzano et al., 2024).

GBA is a membrane-bound lysosomal enzyme responsible for glucosylceramide and glucosylsphingosine hydrolysis. When this enzyme is mutated and dysfunctional, its substrates progressively accumulate within cells, leading to the typical clinical features of GD, including hepatosplenomegaly, bone defects, anemia, lung disease, and central nervous system (CNS) involvement (Mullin et al., 2019). Macrophages result among the most affected cells, becoming engulfed with undigested materials and recognized as Gaucher cells, rising in spleen, liver, and bone marrow but also in other organs, where they enhance inflammation and organ dysfunction (Mullin et al., 2019).

GD patients often present with neurological manifestations. Accordingly, neuronopathic forms of GD were classified as type 2 and 3, while GD type 1 is usually considered deprived of such symptoms. Nonetheless, more recent studies seem to challenge this classification, as GD type 1 patients are reported to potentially develop motor and non-motor neurological symptoms in the course of the disease. Classical neuronopathic forms of disease involve impaired cognitive and motor development, seizures, and psychiatric disorders (Imbalzano et al., 2024). Typically, neuronal accumulation of GBA substrates is accounted for such symptoms, implying neuroinflammation and astrogliosis in patients and mouse models of the disease. This has triggered an increasing interest concerning potential contributes to the disease from other brain cell populations, including astrocytes and microglia (Soria et al., 2017).

*GBA* mutations are also considered the most common genetic risk factor for Parkinson’s disease (PD), with 3%–25% of affected individuals carrying variants of *GBA* (Hertz et al., 2024). However, the mechanisms underlying the role of GBA in PD pathogenesis remain to be elucidated. Although PD has been primarily described as involving dysfunctional dopaminergic neurons, studies in mouse models showed that conditional ablation of the *Gba* gene in dopaminergic neurons of the substantia nigra is not sufficient to induce neurodegeneration and PD-related α-synuclein accumulation, at difference from *in vitro* monoculture models. This suggests the involvement of other cell types in GBA deficiency, as a potential mechanism (Soria et al., 2017).

The mechanistic bases linking *GBA* mutations to PD pathogenesis are not yet fully understood. A common mechanism shared by GD and PD is the accumulation of GBA lipidic substrates able to interact with α-synuclein and promote its aggregation. Furthermore, mutations in *GBA* might lead to ER stress or impaired autophagy with brain accumulation of autophagic substrates together with α-synuclein aggregates, and even to mitochondrial dysfunction (Hertz et al., 2024)

*GBA* was further suggested as a genetic risk factor in multiple system atrophy (MSA), another neurodegenerative disease within the so-called synucleinopathies. MSA is a progressive neurodegenerative disease characterized by parkinsonism manifestations, autonomic failure, cerebellar ataxia, and other dysfunctions. While the cause of MSA remains still undefined, this disorder was described in some works as a primary oligodendrogliopathy characterized by oligodendroglial α-synuclein inclusions. Although *GBA* mutations were found in MSA patients, a general consensus on its direct link to the disease is still debated, and no major insights were so far provided, nor proven, to explain the mechanisms by which inclusions should form within oligodendrocytes (Tseng et al., 2023).

**Figure 1:**
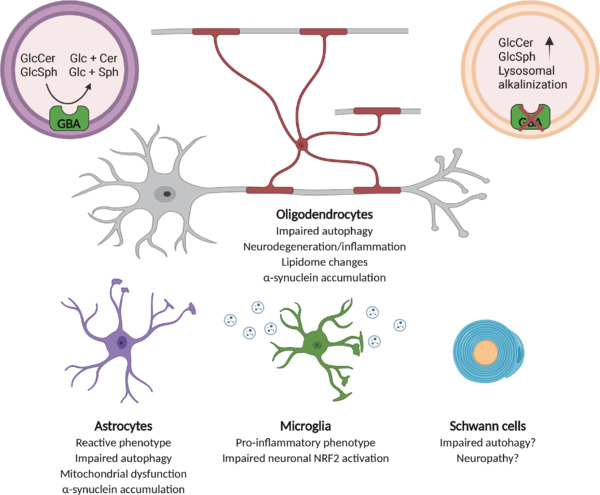
Lack of β-glucocerebrosidase (GBA) in glial cells results in multiple non-overlapping alterations. GBA resides on the luminal membrane side of lysosomes. At acidic pH, it catalyzes the hydrolysis of glucosylceramide (GlcCer) and glucosylsphingosine (GlcSph) to glucose (Glc) and ceramide (Cer) or sphingosine (Sph) respectively. Depletion of GBA leads to accumulation of GlcCer and GlcSph in the lysosomes as well as to a consequential increase in their pH. In *in vitro* and *in vivo* models, GBA inactivation has been shown to affect several cellular processes. Lack of oligodendroglial GBA causes autophagic dysfunction, α-synuclein accumulation and lipidomic changes, ultimately affecting neuronal survival. In other glial cells such as astrocytes, lack of GBA activity turns them into reactive astrocytes. This deficiency shares some molecular downstream effects with oligodendrocytes, as autophagic and mitochondrial dysfunction are reported together with α-synuclein accumulation. Microglia, the main immune cell type of the CNS, relies on GBA for its physiological function as well. Indeed, inactivation of GBA activates their pro-inflammatory state and hampers a microglia-mediated NRF2 activation in neurons. Lastly, a so far neglected role for Schwann cell GBA can be hypothesized. These cells, similarly to oligodendrocytes, provide trophic support to axons, but also require autophagy in case of de- and remyelination. Created in BioRender. Russo, L. (2025) https://BioRender.com/2u22ur2.

**Moving beyond the neuronocentric view in GBA-related pathologies:** Despite the years of efforts in clinical and pre-clinical studies regarding PD and neuronopathic GD, the consensus has seldom moved from the dogmatic idea of a neuronocentric onset of disease. In the last decade, studies have been carried out on models of GD or GBA-related PD, aiming at dissecting the pathomolecular basis of these diseases. Unfortunately, their interpretation has been far from trivial due to model heterogeneity, in terms of chemical or genetic induction, even countering knock-in alternatives of well-known *GBA* human mutations to *Gba* knock-out models. Indeed, the models that truly mimic the latest stages of the human disease derive from either chemical ablation of dopaminergic neurons or overexpression of mutated human aggregation-prone α-synuclein (A53T), while much less is known on the early critical steps in disease onset. Because of the newly gathered data regarding non-neuronal cells of the CNS, now we know that virtually all of them can contribute to the onset and progression of these human disorders (**Figure 1**). Microgliosis and astrogliosis were often reported as pathological features observed in brain tissues from such models and *GBA*-related PD patients, thus triggering interests in understanding the differential contribution exerted from microglia and astrocytes in this context.

Of note, a 50% reduction of GBA activity, obtained in mice through the administration of the chemical inhibitor conduritol-b-epoxide was not sufficient to induce canonical hallmarks of PD, such as a decrease in dopaminergic neurons, but it could trigger microglial activation. Instead, the co-administration of conduritol-b-epoxide and 1-methyl-4-phenyl-1,2,3,6-tetrahydropyridine (MPTP), a toxin typically used in animal models to mimic PD, was able to elicit a greater decrease in tyrosine hydroxylase levels than MPTP alone, as well as an increase in the number of α-synuclein-positive dopaminergic neurons in the substantia nigra pars compacta. This suggests that neurons alone are more resilient to GBA inactivation than commonly thought, possibly due to modulatory mechanisms from other cell types (Mus et al., 2019). In line with this, other works showed that cell-specific ablation of *GBA* in microglia does not induce neurodegeneration, but might hinder a protective role towards neurons resulting in a higher vulnerability to toxins (Brunialti et al., 2021).

The inhibition of GBA can also affect astrocytes, key players in the provision of neuronal trophic support and protection, as well as in synapsis modulation. An endo-lysosomal and autophagic dysfunction is found in primary astrocytes from *Gba*^-/-^ mice, similarly to other cell types lacking *Gba*, even leading to mitochondrial dysfunction. Additionally, *Gba* mutant astrocytes derived from knock-in PD murine models are characterized by a dysregulated inflammatory response, a reduced lysosomal protease activity, while their ability to degrade α-synuclein appears intact. In more translational approaches, GD patients-derived iPSCs differentiated into astrocytes displayed extensively reactive features and cytoskeletal hypertrophy, together with impaired lysosomal activity and α-synuclein accumulation, supporting the idea that altered astrocytes, might have a role in exacerbating neurodegeneration (Kam et al., 2020).

Overall, our understanding of glial contribution to GBA-related PD remains limited, but the use of cell-specific knockout models can help to untangle the single contribution of every cell type when looking at this disorder.

**Why should white matter be on focus in GBA deficiencies?** In the context of GBA deficiencies, oligodendrocytes have received comparatively little attention, in contrast to other glial cells, but growing evidence suggests that myelinating cells will no longer be neglected in GD and PD. Indeed, the presence of white matter microstructural alterations was recently demonstrated in the CNS of pediatric GD type 1 patients. As well, fiber-specific white matter alterations were described in non-medicated early-stage PD patients bearing *GBA* mutations, triggering an interest in understanding oligodendroglial involvement in the pathology. In parallel, the onset of peripheral neuropathy was reported in GD patients, showing lipid aggregates within Schwann cells and axons (Imbalzano et al., 2024). Furthermore, although PD has been primarily described as involving dysfunctional dopaminergic neurons due to α-synuclein accumulation, recent studies suggested that white matter alterations could sustain PD pathogenesis and may even play a role in disease onset (Yang et al., 2023). Based on these premises, we decided to test the impact of GBA loss of function specifically in myelinating cells, by producing a conditional knockout mouse line, named *Gba1*^*fl/fl*^*::Cnp1*^*Wt/cre*^. This mouse line bears a deletion of the exons encoding the catalytic domain of β-glucocerebrosidase exclusively in oligodendrocytes and Schwann cells.

Our research focused on the CNS and highlighted the onset of demyelination in adult mice, with a reduction of myelin-related protein and lipid levels accompanied by increased g-ratio in large caliber axons of striatum, optic nerve, and cerebellum. Such alterations were not linked to a loss of oligodendrocytes, but rather to a reduced differentiation, as shown also *in vitro* by *Gba* knockout primary oligodendrocytes, when compared to wild-type ones (Gregorio et al., 2024). More interestingly, our data demonstrated the presence of an altered brain lipidomic profile, supporting the idea that GBA ablation in oligodendrocytes impacts globally on brain lipid metabolism. Moreover, an early onset of neurodegenerative hallmarks, such as axonal degeneration, astrogliosis, and increased density of inflammatory Iba1-positive cells, accompanied by mild behavioral deficits, was recorded in *Gba1*^*fl/fl*^*::Cnp1*^*Wt/cre*^ CNS regions when compared to control mice (Gregorio et al., 2024).

Notably, in the animal model we have recently characterized, we were able to highlight α-synuclein aggregation and neurodegeneration, despite the lack of a GBA defect in neuronal cells. These results are consistent with the existence of non-cell autonomous drivers of PD-related features that might be originated or pushed forward by non-neuronal cells, including oligodendrocytes. One possibility is that neurons might rely on oligodendroglial cells for the degradation of GBA substrates, which can be transferred from one cell type to the other, as suggested by a study by Wang et al. (2022).

**Conclusions:** To better elucidate the mechanisms underlying the defects we observed, and the processes primarily altered in oligodendrocytes upon GBA inactivation, further studies should be developed. Nonetheless, an impairment of the autophagic activity, a common feature already highlighted in other cell types, emerged also in oligodendroglial cell lines. Indeed, conduritol-b-epoxide-treated Oli-neu cells displayed reduced acidification in lysosomes leading to impaired autophagic and lysosomal degradation, manifested through the accumulation of α-synuclein and autophagic cargo proteins (Gregorio et al., 2024).

Besides the overlooked role of oligodendrocytes in GBA deficiencies, an even less explored phenotype is the presence of peripheral nervous system defects in non-neuronopathic GD. Considering both the increasing knowledge on the involvement of myelinating glia in GBA-PD and the high incidence of peripheral neuropathy in GD patients, the altered myelination observed in the CNS of the *Gba1*^*fl/fl*^*::Cnp1*^*Wt/cre*^ mouse line might be shared by Schwann cells in the peripheral nervous system (Chérin et al., 2010; Imbalzano et al., 2024). In these regards, our efforts are now focusing on the study of the peripheral nervous system phenotype in our conditional knockout mouse model. The expected impairment in the autophagic and lysosomal clearance in Schwann cells might indeed have a role in the proper development of peripheral nerves and in the accomplishment of peripheral motor and sensory functions. Of note, myelinophagy, a Schwann cell–specific form of autophagy triggered by nerve injury, aimed at degrading myelin membranes, is determinant in the concomitant reprogramming of myelinating Schwann cells into repair cells, contributing to nerve regeneration (Gomez-Sanchez et al., 2015). Therefore, understanding whether GBA plays a major role in guaranteeing Schwann cell physiology, will not only open to novel knowledge and therapeutic strategies for PD/GD-related neuropathies, but could provide a novel target to sustain nerve regenerative processes dependent on Schwann cell degradative abilities.

*This work was funded by the AFM-Telethon Foundation (#28703) and by the Italian Ministry of Education, University and Research (Grant P2022Y2A3L funded in the framework of NRRP, Mission 4.2, Investment 1.1 “progetti di ricerca di Rilevante Interesse Nazionale ‐ PRIN”, funded by the European Union ‐ Next Generation EU, CUP C53D23007520001; Grant P20227YB93, CUP C53D23003030001) (to MC). Moreover, as part of the activities of the National Center for Gene Therapy and Drugs based on RNA Technology, funded in the framework of the National Recovery and Resilience Plan (NRRP), Mission 4 “Education and Research”, Component 2 “From Research to Business”, Investment 1.4 “Strengthening research structures for supporting the creation of National Centres, national R&D leaders on some Key Enabling Technologies”, this work was funded by the European Union ‐ Next Generation EU, Project CN00000041, CUP B93D21010860004, Spoke n. 5 “Inflammatory and infectious diseases” (to MC).*
